# Supplementary material for: The association between perceived sensitivity to medicines, reported side effects and personal characteristics: A secondary analysis of an RCT
Source: PLoS One. 2024 Oct 21;19(10):e0308213. doi: 10.1371/journal.pone.0308213 (PMC11493249; doi:10.1371/journal.pone.0308213)
Supplement: S1 Table — (DOCX) [file pone.0308213.s001.docx]

Supplementary table a.

Spearman coefficients for correlations between PSM (independent variable), side effect scores (dependent variable) and nonparametric variables.


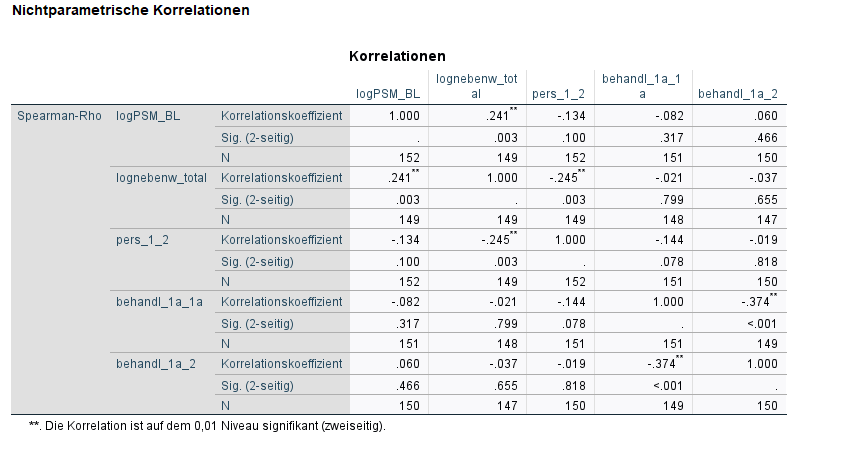


log = log transformed, BL = baseline, PSM = Perceived Sensitivity to Medicines, nebenw_total = total side effect scores, pers_1_2 = gender (male/female), behandl_1a_1a = medical care utilization (yes/no), behandl_1a_2 = nonmedical care utilization (yes/no)

Supplementary table b.

Pearson coefficients for correlations between PSM (independent variable), side effect scores (dependent variable) and parametric variables.
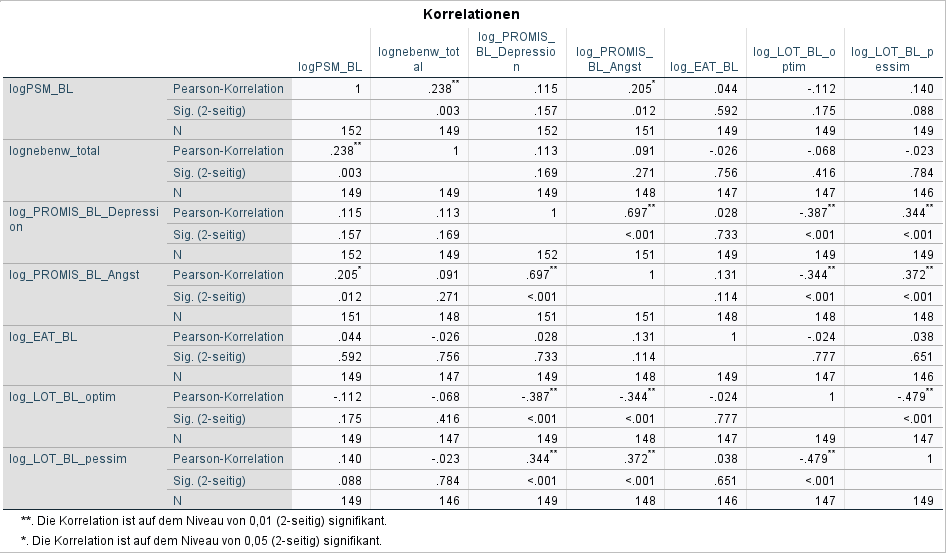


log = log transformed, BL = baseline, PSM = Perceived Sensitivity to Medicines, nebenw_total = total side effect scores, PROMIS = Patient-Reported Outcome Measurement Information System , Angst = anxiety , EAT = expectation , LOT-R = Life Orientation Test Revised , optim = optimism , pessim = pessimism
