# Supplementary material for: The association between perceived sensitivity to medicines, reported side effects and personal characteristics: A secondary analysis of an RCT
Source: PLoS One. 2024 Oct 21;19(10):e0308213. doi: 10.1371/journal.pone.0308213 (PMC11493249; doi:10.1371/journal.pone.0308213)
Supplement: Attachment — Submitted filename: Response_to_Reviewers_20240626.docx [file pone.0308213.s003.docx]

**Response to Reviewers**

Reviewer #1: Congratulation. The paper is ready for the publication.
I am more than happy to accept it.
Please continue to write this interesting ma uscito.

**Response**: Thank you very much for diligently reviewing the manuscript and for your words of encouragement.

Reviewer #2: The authors are very responsive on the critiques. However, some responses are only in the rebuttal letter, but not reflected in the manuscript. Please keep in mind the audience don’t get the chance to read to rebuttal letter. This is particular relevant to my previous comment 5, a sensitivity analysis by looking at the side effects scores at earlier and later sessions.

**Response:** Thank you for this valuable note. In consequence, we included a comment on the sensitivity analysis in the result section of the manuscript, which reads: In an additional sensitivity analysis we looked at the side effect score of the first four sessions. Our analysis revealed the same baseline variables as predictors of side effects compared to the predictive baseline variables across all eight sessions.

On the pairwise correlation tables, first it is more proper to be named Supplementary tables than appendices since there is analysis involved; second, it is not referred to in the main text.

**Response:** This is correct, thank you. It is indeed a supplement not an appendix. We renamed the file accordingly. Furthermore, we referred to the tables in the result section of the manuscript as follows: Tables with pairwise correlations can be found in the supplementary tables.
